# Cathepsin B as a potential prognostic and therapeutic marker for human lung squamous cell carcinoma

**DOI:** 10.1186/1476-4598-12-125

**Published:** 2013-10-20

**Authors:** Fengming Gong, Xingchen Peng, Can Luo, Guobo Shen, Chengjian Zhao, Liqun Zou, Longhao Li, Yaxiong Sang, Yuwei Zhao, Xia Zhao

**Affiliations:** 1 Department of Gynecology and Obstetrics, Second West China Hospital, and State Key Laboratory of Biotherapy and Cancer Center, West China Hospital, Sichuan University, Chengdu, China; 2 Department of Medical Oncology, Cancer Center, State Key Laboratory of Biotherapy, West China Hospital, Sichuan University, Chengdu, China; 3 State Key Laboratory of Biotherapy and Cancer Center, West China Hospital, West China Medical School, Sichuan University, Chengdu, China

**Keywords:** Lung squamous cell carcinoma, 2-DE, Cathepsin B, Shh, Ptch

## Abstract

**Background:**

The lung squamous cell carcinoma survival rate is very poor despite multimodal treatment. It is urgent to discover novel candidate biomarkers for prognostic assessment and therapeutic targets to lung squamous cell carcinoma (SCC).

**Results:**

Herein a two-dimensional gel electrophoresis and ESI-Q-TOF MS/MS-based proteomic approach was used to identify differentially expressed proteins between lung SCC and adjacent normal tissues. 31 proteins with significant alteration were identified. These proteins were mainly involved in metabolism, calcium ion binding, signal transduction and so on. Cathepsin B (CTSB) was one of the most significantly altered proteins and was confirmed by western blotting. Immunohistochemistry showed the correlation between higher CTSB expression and lower survival rate. No statistically significant difference between CTSB-shRNA treated group and the controls was observed in tumor volume, tumor weight, proliferation and apoptosis. However, the CTSB-shRNA significantly inhibited tumor metastases and prolonged survival in LL/2 metastatic model. Moreover, CTSB, Shh and Ptch were up-regulated in patients with metastatic lung SCC, suggesting that hedgehog signaling might be activated in metastatic lung SCC which could affect the expression of CTSB that influence the invasive activity of lung SCC.

**Conclusions:**

These data suggested that CTSB might serve as a prognostic and therapeutic marker for lung SCC.

## Introduction

Lung cancer is the most common cancer in both men and women, with deaths in 2012 estimated to exceed 157,300 in the United States [1]. Lung squamous cell carcinoma (SCC) is the most common type, accounting for about 30-50% of all lung cancers patients [2]. The survival rate of lung SCC remains low, though improvements in surgery, radiotherapy and chemotherapy were made [2]. Therefore, it is crucial to discover novel biomarkers for early detection of lung SCC and monitoring of disease progression. Furthermore, the identification of novel therapeutic targets would also facilitate drug development for lung SCC.

The two-dimensional gel electrophoresis (2-DE) based proteomics approach provides a powerful tool to analyze the expression levels of proteins in tissue samples. This may enable the identification of cancer related proteins for early diagnosis, therapeutic intervention and prognostic assessment [3,4]. Several papers have carried out proteomic studies of lung SCC and some proteins have been identified [5-7]. However, few biomarkers for lung SCC have been introduced into clinical use because of insufficient validation and the absence of prospective studies. Moreover, there is a lack of the fundamental understanding required for clinical applications and need a better comprehension of the underlying biological processes.

Tumorigenesis is a complex process that involves equal contribution from extracellular as well as intracellular proteolytic events [8]. Invasion and metastasis of cancer result from several interdependent processes in which proteolytic enzymes have been implicated [9]. Cathepsins B (CTSB), composed of a heavy chain of 25–26 kDa and a light chain of 5 kDa, is a family of lysosomal cysteine proteases and plays an important role in intracellular proteolysis [10]. Activity of CTSB is known to be important for tumorigenesis, angiogenesis, invasion and metastasis [11,12]. Elevated levels of CTSB expression have been reported in prostate cancer [13], colorectal cancer [14], gliomas [15], melanomas [16], breast cancer [17,18]. However, few papers demonstrated that CTSB was over-expressed in lung SCC by proteomics.

To the best of our knowledge, correction of CTSB expression in lung SCC with prognosis and therapy has yet to be determined. Moreover, the previous study subject mainly consisted of Caucasian while only a few proteomic studies on lung SCC of Asian population have been reported. The ethnic differences among patients may contribute to different findings. This study aimed to determine whether expression levels of CTSB in lung SCC of Asian population could affect proliferation, invasion, metastasis and prognosis. Furthermore, possible molecular mechanism was investigated.

## Materials and methods

### Clinical specimens

Fresh human lung SCC and paired adjacent normal tissues were obtained from 8 patients suffering lung SCC who underwent surgical resections. Primary human lung SCC tissues were obtained from 8 patients suffering metastatic lung SCC by bronchoscopy. The specimens were diagnosed histological after staining with H&E, and the surgical pathologic stage was determined according to the TNM classification system of the International Union against Cancer. Detailed information of the patients was shown in Table 1. All pairs of samples were immediately frozen in liquid nitrogen prior to experiments. This study was approved by the Institutional Ethics Committee of Sichuan University and informed consents were obtained from all patients prior to analysis.

**Table 1:** The clinical and pathologic data of patients with lung SCC for 2-DE

**Sample No**	**Gender**	**Age**	**Histological type**	**TNM classification**	**Clinical stage**
1	Male	58	Poor diff.	T2bN1M0	IIb
2	Male	61	Mod diff.	T1N1M0	IIa
3	Female	59	Poor diff.	T3N1M0	IIIa
4	Male	71	Mod diff.	T2aN0M0	Ib
5	Male	49	Well diff.	T1N0M0	Ia
6	Female	53	Poor diff.	T3N1M0	IIIa
7	Male	66	Well diff.	T1N1M0	IIa
8	Female	49	Mod diff.	T2aN1M0	IIa

### Two-dimensional electrophoresis and image analysis

Tissues were ground into powder in liquid nitrogen and lysed in 1 ml lysis buffer (7 M urea, 2 M thiourea, 4% CHAPS, BioRad, USA) containing protease inhibitor cocktail 8340 (Sigma, St Louis, MO, USA). Samples were then kept on ice and sonicated in five cycles of 10 s, each consisting of 5 s sonication followed by a 10 s break. After centrifugation at 15000 rpm for 1 h at 4°C, the supernatant was collected and the protein concentrations were determined using the DC protein assay kit (Bio-Rad). Protein samples (2 mg) were applied to IPG strip (17 cm, pH3-10NL, Bio-Rad) using a passive rehydration method. After 16 h of rehydration, the strips were transferred to an IEF Cell (Bio-Rad). IEF was performed as follows: 250 V for 30 min, linear; 1000 V for 1 h, rapid; linear ramping to 10000 V for 5 h, and finally 10000 V for 5 h [19]. The second dimension was performed using 12% SDS-PAGE at 30 mA constant current per gel after equilibration [20]. The gels were stained using CBB R-250 (Merck, Germany) and scanned with a Bio-Rad GS-800 scanner. Four independent runs were made for each sample to ensure the accuracy of analyses. The maps were analyzed by PDQuest software Version 6.1 (Bio-Rad). The quantity of each spot in a gel was normalized as a percentage of the total quantity of all spots in that gel and evaluated in terms of OD. Paired *t*-test was performed to compare data. Only spots that showed significant differences (± over two fold, P < 0.05) were selected for analysis with MS.

### In-gel digestion

In-gel digestion of proteins was carried out using MS grade Trypsin Gold (Promega, Madison, WI) according to the manufacturer’s instructions. Briefly, spots were cut out of the gel (1-2 mm diameter) using a razor blade, and destained twice with 100 mM NH_4_HCO_3_/50% ACN at 37°C for 45 min in each treatment. After drying, the gels were preincubated in 10-20 μl trypsin solution for 1 h. Then, 15 μl digestion buffer was added (40 mM NH_4_HCO_3_/10% ACN) to cover gel and incubated overnight at 37°C. Tryptic digests were extracted using MilliQ water initially, followed by twice extraction with 50% ACN/5% TFA for 1 h each time. The combined extracts were dried in a vacuum concentrator at room temperature. The samples were then subjected to mass spectrometry (MS) analysis.

### Electrospray ionization quadrupole time-of-flight(ESI-Q-TOF) analysis and protein identification

Mass spectra were acquired using a Q-TOF-MS (Micromass, Manchester, UK) fitted with an ESI source (Waters). Tryptic digests were dissolved in 18 μl 50% ACN. MS/MS was performed in a data-dependent mode in which the top ten most abundant ions for each MS scan were selected for MS/MS analysis. Trypsin autolysis products and keratin derived precursor ions were automatically excluded.

The MS/MS data were acquired and processed using MassLynx software (Micromass) and MASCOT (http://www.matrixscience.com) was used to search the database. Database searches were carried out using the following parameters: Database, Swiss-Prot; taxonomy, *homo sapiens*; enzyme, trypsin; mass tolerance, ± 0.1 Da; MS/MS tolerance, ± 0.05 Da; and an allowance of one missed cleavage. Fixed modifications of cysteine carboamidomethylation, and variable modifications of methionine oxidation were allowed. The data format was selected as Micromass PKL and the instrument was selected as ESI-Q-TOF. Proteins with probability based MASCOT scores exceeding their threshold (P < 0.05) were considered to be positively identified. To eliminate the redundancy of proteins appearing in the database under different names or accession numbers, the one-protein member with the highest MASCOT score,and belonging to the species *Homo sapiens*, was further selected from the relevant multiple-member protein family.

### Real time RT-PCR

mRNA was isolated from frozen tumor tissue using RNeasy total RNA kits (Qiagen, Hilden, Germany). RNA concentration was evaluated by photometric measurement at 260/280 nm. 1 μg RNA was used for cDNA synthesis using the QuantiTect Reverse Transcription Kit (Qiagen, Hilden, Germany). PCR reactions were performed by using the Platinum® SYBR® Green qPCR SuperMix-UDG (Invitrogen, Karlsruhe, Germany) in the 7300 real-time PCR system (Applied Biosystems, Darmstadt, Germany). The paired forward and reverse primers:CTSB (forward) 5′-CCGCTCGAGGCCACCTGGCAGCTCTGGGCCTCCCT-3′, and (reverse) 5′-ACGCGTCGACTTAGATCTTTTCCCAGT-3′; Shh (forward) 5′-GATGTC TGCTGCTAGTCCTCG-3′, and (reverse) 5′-CACCTCTGAGTCATCAGCCTG-3′; Ptch (forward) 5′-CCACAGAAGCGCTCCTACA-3′, and (reverse) 5′-CTGTAATTTCGCCCCTT CC-3′. The experiment was repeated three times.

### Western blotting assay

Proteins from tissues or cells were extracted in RIPA buffer (50 mM Tris-base, 1.0 mM EDTA, 150 mM NaCl, 0.1% SDS, 1% Triton X-100, 1% Sodium deoxycholate, 1 mM PMSF) and quantified by the DC protein assay kit (Bio-Rad). Samples were separated by 12% SDS-PAGE and transferred to PVDF membranes (Amersham Biosciences). The membranes were blocked overnight with PBS containing 0.1% Tween 20 in 5% skimmed milk at 4°C, and subsequently probed by the primary antibodies: anti-CTSB (Biovisin Res, USA), Anti-Shh and anti-Ptch (Santa Cruz Biotechnology, Germany). Blots were incubated with the respective primary antibodies for 2 h at room temperature and washed three times in TBST. After that, the blots were incubated with secondary antibody conjugated to HRP for 2 h at room temperature. Target proteins were detected by enhanced chem-iluminescence reagents (Amersham Pharmacia Biotech, Piscataway, USA). β-actin was used as an internal loading control. The experiment was repeated three times.

### Immunohistochemistry

The sections were stained by the Envision System-HRP method (DakoCytomation Inc, Carpinteria, CA), according to the kit manufacturer’s instructions. Specific antibodies performed included anti-human CTSB and anti-human PCNA (Santa Cruz Biotechnology, Germany). For each section, a minimum of 5 representative fields with well-preserved carcinoma tissue was examined at × 400 magnifications (Olympus Optical, Japan), and 200 carcinoma cells were counted for each field. An average for immune-staining intensity or percentage of positive cells was taken over these fields. In statistical analysis, with reference to Jeffrey’s study [21], staining of CTSB was scored as the product of the staining intensity (on a scale of 0–3: negative = 0, weak = 1, moderate = 2, strong = 3) × the percentage of cells stained (positively recorded on an ordered categorical scale: 0 = zero, 1 = 1-25%, 2 = 26-50%, 3 = 51-100%), resulting in a scale of 0–9. The evaluation was performed by two independent investigators, without any prior knowledge of each patient’s clinical information. Any discrepancy between the two evaluators was resolved by reevaluation and careful discussion until agreement was reached.

### shRNA plasmid vector construction

shRNA targeting human CTSB were purchased from Santa Cruz Biotechnology, Germany . The HK sequence, which has no homology with any mammalian sequence, was used as negative control (NC group). Plasmids were extracted using a Qiagen Plasmid Mega Kit (Qiagen, Hilden, Germany) and stored at -20°C.

### Cell culture and transfection

Human lung carcinoma cell line A549 and mouse Lewis lung carcinoma cell line LL/2 (ATCC) were maintained in RPMI 1640 or DMEM medium. The lipofectamine 2000 (Invitrogen) and shRNA were diluted in antibiotics free media, respectively, and then combined at a ratio of 2.5:1. Cells were transfected in indicated concentrations according to the manufacturer’s recommendation.

### Tumor xenograft model and shRNA treatment

Healthy female nude mice (6–8 weeks) were injected subcutaneously with A549 cells (5 × 10^6^ / 100 μl PBS per mouse) via the right flank. After 7 days, when the tumor diameters were about 0.6 cm, the mice were randomly divided into four groups (seven per group) for caudal vein injections. The groups were as follows: (1) PBS, 100 μl of PBS; (2) Lipo, lipofectamine 2000 62.5 μg/100 μl of PBS; (3) Negative control, Pgenesil-2-HK-shRNA 25 μg/100 μl of PBS; (4) shRNA, Pgenesil-2-CTSB-shRNA 25 μg/100 μl of PBS. Caudal vein injections were performed every three days, and tumor volumes were evaluated according to the following formula: tumor volume(mm^3^) = 0.52 × length × width^2^. The dissected tumors were fixed in neutral buffered formalin and embedded in paraffin, and sections (5 μm) were stained with H&E. The animal experiment was repeated three times.

### TUNEL assay

Apoptotic cells within the tumor sections were evaluated by the terminal deoxynucleotidyl transferase-mediated dUTP nick-end labeling (TUNEL) technique. Percent apoptosis was determined by counting the number of apoptotic cells and dividing by the total number of cells in the field (5 high power fields/slide).

### Therapy of lung metastatic models

Female C57BL/6 mice (6–8 weeks) were purchased from experimental animal center of Sichuan University (Chengdu, Sichuan province, China) and were housed in our animal research facility. Each mouse was inoculated with LL/2 cells (5 × 10^5^) via the caudal vein to establish lung metastatic model. These lung metastatic mice were randomly assigned into the following four groups at day 12 and each mouse received the corresponding treatment by caudal vein injection: (1) PBS, 100 μl of PBS; (2) Lipo, lipofectamine 2000 62.5 μg/100 μl of PBS; (3) Negative control, Pgenesil-2-HK-shRNA 25 μg/100 μl of PBS; (4) shRNA, Pgenesil-2-CTSB-shRNA 25 μg/100 μl of PBS. Caudal vein injections were performed every three days. After 6 mice from each group were sacrificed at day 30, lung net weight of each mouse was measured. Autopsy was performed to determine the number of the metastatic nodules of lung. The other mice (ten mice/group) were followed for survival time. The animal experiment was repeated three times.

### Matrigel invasion assay

Cells were trypsinized and counted, after 48 h transfection of A549 cells with PBS, Lipo, negative control and CTSB-ShRNA. Cells (1 × 10^5^) were counted using a hemocytometer and cultured in the upper chamber of a transwell insert (8-μM pores) coated with matrigel (1 mg/mL) (Collaborative Research Inc., Boston, MA) in the presence of 500 μl serum-free media. 700 μl serum-supplemented media added to the lower chamber served as a chemo-attractant and the chambers were maintained in an incubator at 37°C. After a 48 h incubation period, the chambers were removed from the incubator, non-migrated cells in the upper chamber were scraped, and migrated cells adhering to the lower surface of transwell insert were stained with crystal violet. Photographs of the cells were taken at a 200 magnification with a light microscope. The cells were counted.

### Data analysis and statistics

Paired t-test and one way ANOVA was used to analyze differences between groups. Survival curves were generated according to the Kaplan-Meier method and the statistical analyses were performed using log-rank test. Relevance analysis of ordinal data was performed by cross x^2^ test. P < 0.05 was considered significant in all analyses.

## Results

### Differentially expressed proteins between lung SCC and adjacent normal tissues

**Figure 1:**
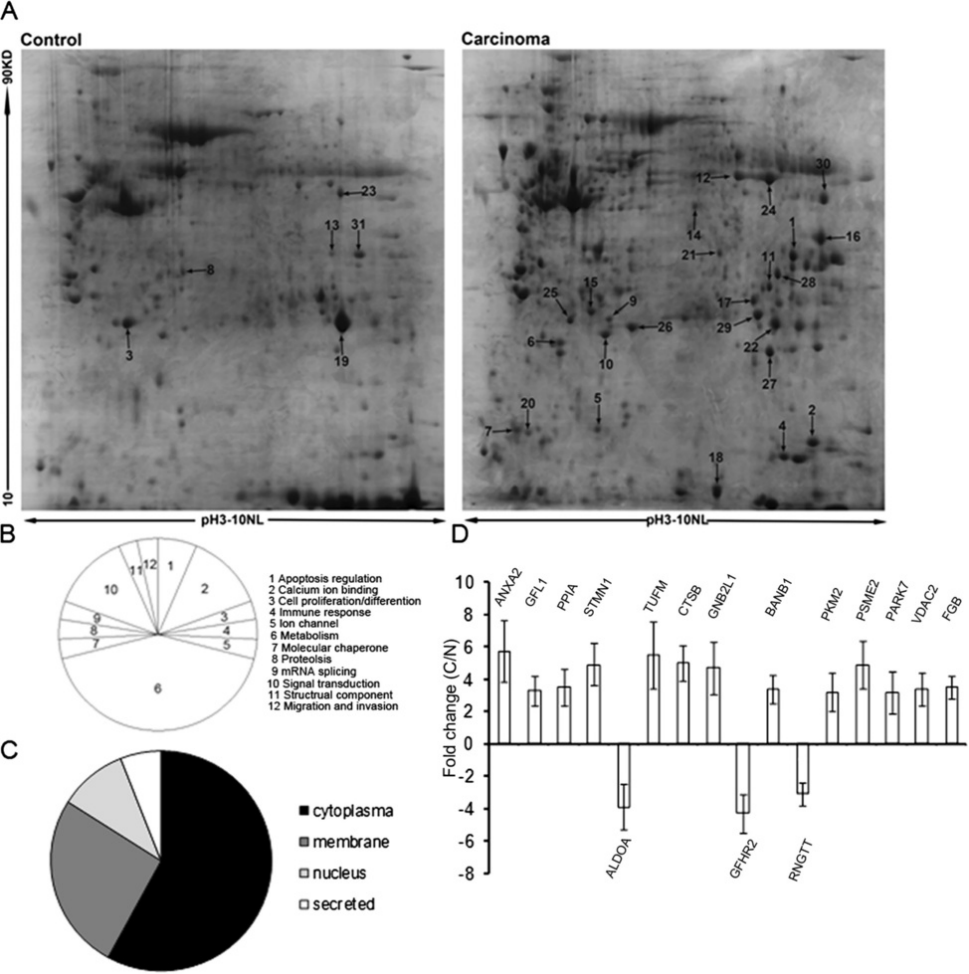
**Representative 2-DE gel images of lung SCC and adjacent normal tissues. (A)** Fresh human lung SCC and paired adjacent normal tissues of Asian population were obtained from 8 patients suffering lung SCC who underwent surgical resections. Total protein extracts were separated on pH 3–10 nonlinear IPG strips in the first dimension followed by 12% SDS-PAGE in the second dimension and visualized by CBB staining. The arrows indicate the 31 differentially expressed proteins. **(B)** Cluster analysis of the changed proteins revealed that the altered proteins were involved in different biological processes, including metabolism (40%), calcium ion binding (13%), signal transduction (13%) and so on. **(C)** The identified proteins were categorized into four groups according to their subcellular locations. 58% of the total proteins were located in the cytoplasm, and the remainder was situated in the nuclear (26%), cell membrane (10%) and secreted protein (6%). **(D)** Expression profile of the 16 altered proteins (3-fold change) as shown in Figure 1A. The intensities of spots were quantified using PDQuest 2-D analysis software.

**Figure 2:**
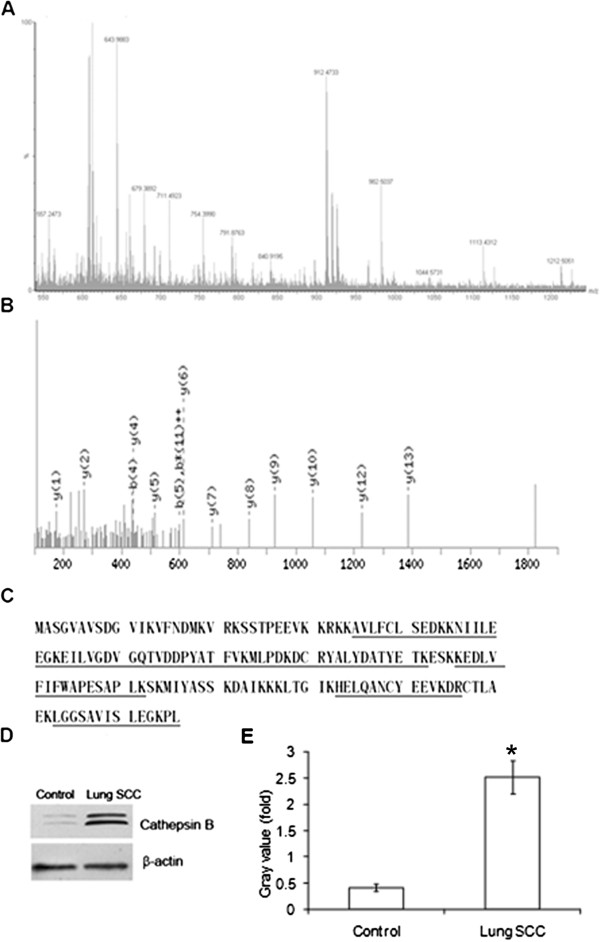
**CTSB as the representative of protein identification using ESI-Q- TOF-MS/MS. (A** &**B)** Mass spectrogram of tryptic peptides from spot #15. **(C)** Protein sequence of CTSB. The matched peptides are underlined. **(D)** Western blotting confirmation of the proteins CTSB, corresponding to the spot #15. As shown, expression of CTSB was up-regulated in lung SCC compared with adjacent normal tissues. The experiment was repeated three times. **(E)** The level of CTSB was 2.52 ± 0.31 in the lung SCC group, and 0.42 ± 0.07 in control (adjacent normal tissues) (P < 0.05). β-actin was used to normalize for any differences in protein loading between lanes.

2-DE was performed with lung SCC and adjacent normal tissues from 8 patients. Image analysis was performed using PDQuest 6.1 software, and displayed well-resolved and reproducible protein profiles for both lungs SCC and adjacent normal tissues (Figure 1A). 31 spots were selected and analyzed using ESI-Q-TOF MS/MS. Because different isoforms of a protein might have distinct functions, each isoform/spot was considered to be a single protein for analysis in our study. Cluster analysis revealed that the altered proteins were involved in different biological processes, including metabolism (40%), calcium ion binding (13%), signal transduction (13%) and so on (Figure 1B). Data of details was listed in Table 2. The identified proteins were categorized into four groups according to their sub-cellular locations. 58% of the total proteins were located in the cytoplasm, and the remainder was situated in the nuclear (26%), cell membrane (10%) and secreted protein (6%) (Figure 1C). Expression profile of the 16 altered proteins (3-fold change) was shown in Figure 1D. Among them, CTSB was identified with significant alteration. It was up-regulated 5.0-fold in tumor compared with pair adjacent normal tissue (P < 0.05). Furthermore, ESI-Q-TOF-MS/MS analysis revealed that CTSB has 11 matched peptides and a MASCOT score of 144, as shown in Figure 2A, B, C.

**Table 2:** Identified proteins by MS/MS analysis

**Spot No.**	**Protein description**	**Gene name**	**Function**	**Accession no.**	**Theoretical Mr/pI**	**Score**	**No. of pep**	**Fold change**
1	Annexin A2	ANXA2	Calcium ion binding	P07355	38472/7.56	518	19	↑5.71 ± 1.9
2	Cofilin-1	CFL1	Rho protein signal transduction	P23528	18371/8.26	563	32	↑3.3 ± 0.9
3	Apolipoprotein A-I	APOA1	Metabolism	P02647	28078/5.27	632	28	↓2.3 ± 0.9
4	Peptidyl-prolyl cis-trans isomerase A	PPIA	Metabolism	P62937	17881/7.82	335	19	↑3.5 ± 1.1
5	Stathmin	STMN1	Signal transduction	P16949	17171/5.77	84	4	↑4.9 ± 1.3
6	Calcium binding protein P22	CHP	Calcium ion binding	Q99653	22442/4.98	37	2	↑2.1 ± 0.2
7	Myosin regulatory light chain 12A	MYL12A	Calcium ion binding	P19105	19662/4.67	147	9	↑2.4 ± 0.6
8	Annexin A4	ANXA4	Calcium ion binding	P09525	35751/5.85	1024	39	↓2.2 ± 0.5
9	Heat shock protein beta-1	HSPB1	Molecular chaperone	P04792	22782/5.98	168	10	↑2.3 ± 0.8
10	Glutathione S-transferase P	GSTP1	Apoptosis regulation	P09211	23224/5.44	342	22	↑2.1 ± 0.4
11	Glyceraldehyde-3-phosphate dehydrogenase	GAPDH	Metabolism	P04406	35922/8.58	952	36	↑2.1 ± 0.7
12	Beta-enolase	ENO3	Cell proliferation/differentiation	P13929	46800/7.73	267	10	↑2.5 ± 1.2
13	Fructose-bisphosphate aldolase A	ALDOA	Metabolism	P04075	39288/8.39	406	25	↓3.9 ± 1.4
14	Elongation factor Tu, mitochondrial	TUFM	Metabolism	P49411	49858/7.26	662	33	↑5.5 ± 2.1
15	Cathepsin B	CTSB	Migration and invasive	P07858	27814/5.23	144	11	↑5.0 ± 1.1
16	RACK1	GNB2L1	Metabolism	P63244	35055/7.60	205	5	↑4.7 ± 1.6
17	PDZ and LIM domain protein 1	PDLIM1	Structrual component	O00151	36513/6.56	131	14	↑2.1 ± 0.3
18	Acyl-CoA-binding protein	DBI	Metabolism	P07108	10038/6.12	220	5	↑2.3 ± 0.4
19	Complement factor H-related protein 2	CFHR2	Metabolism	P36980	27896/6.52	61	10	↓4.3 ± 1.2
20	Apoptosis regulator BAX	BAX	Apoptosis regulation	Q07812	21184/5.08	91	9	↑2.4 ± 0.8
21	HLA class I histocompatibility antigen, A-25 alpha chain	HLA-A	immune response	P18462	38707/5.97	24	5	↑2.2 ± 0.5
22	BMP and activin membrane-bound inhibitor homolog	BAMBI	Signal transduction	Q13145	25956/8.21	255	12	↑3.4 ± 0.9
23	mRNA-capping enzyme	RNGTT	mRNA splicing	O60942	52533/8.3	44	9	↓3.1 ± 0.7
24	Pyruvate kinase isozymes M1/M2	PKM2	Metabolism	P14618	57805/7.95	120	14	↑3.2 ± 1.2
25	Proteasome activator complex subunit 2	PSME2	Proteolysis	Q06323	27515/5.44	327	17	↑4.9 ± 1.5
26	Protein DJ-1	PARK7	Signal transduction	Q99497	20050/6.33	566	16	↑3.2 ± 1.3
27	Transgelin	TAGLN	Metabolism	Q01995	22653/8.87	369	4	↑2.8 ± 0.6
28	Glyceraldehyde-3-phosphate dehydrogenase	GAPDH	Metabolism	P04406	35922/8.58	345	10	↑2.3 ± 0.5
29	Voltage-dependent anion-selective channel protein 2	VDAC2	Ion channel	P45880	38068/6.33	47	3	↑3.4 ± 1.0
30	Fibrinogen beta chain precursor	FGB	Metabolism	P02675	55928/7.95	55	14	↑3.5 ± 0.7
31	Creatine kinase B-type	CKB	Metabolism	P12277	42644/5.35	155	9	↓2.4 ± 0.8

Upward arrows: Up-regulated.

Downward arrows: Down-regulated.

### Over-expression of CTSB in lung SCC

To confirm the altered expression of CTSB in lung SCC, western blotting analysis was performed using anti-CTSB antibody, and over-expression of CTSB was observed in the carcinoma tissues examined (carcinoma tissues: 2.52 ± 0.31; adjacent normal tissues: 0.42 ± 0.07; Student’s t test, P < 0.05) (Figure 2D, E). Taken together, our data demonstrated that CTSB was over-expressed in lung SCC at the protein level, which was consistent with the observation made in the 2-DE analysis.

### Over-expression of CTSB was correlated with poor prognosis

**Figure 3:**
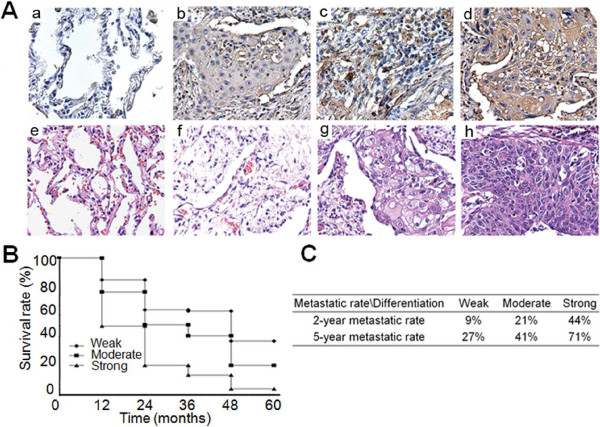
**IHC analysis of CTSB expression in lung SCC and adjacent normal tissues. (A)** Staining against CTSB showed significant differences between lung SCC and adjacent normal tissues, and overexpression of CTSB was likely to present with bad differentiation. IHC (a: adjacent normal tissues; b: well differentiated, weakly positive; c: moderately differentiated, moderately positive; d: poorly differentiated, strongly positive.); H & E (e: adjacent normal tissues; f: well differentiated; g: moderately differentiated; h: poorly differentiated). **(B)** Kaplan-Meier curves and statistics showed the correlation between CTSB expression and decreased survival. The 5-year survival rates were 41%, 20% and 2% for weak, moderate and strong staining samples, respectively, when CTSB was assessed as a marker (Statistical difference: weak VS moderate: P = 0.041; weak VS strong: P = 0.009; moderate VS strong: P = 0.021). Multivariate analyses using Cox proportional hazard model also showed that CTSB could also be developed as a prognostic factor for lung SCC. **(C)** Patient data showed the 2-year metastatic rates were 9%, 21% and 44% for weak, moderate and strong staining samples; the 5-year metastatic rates were 27%, 41% and 71% for weak, moderate and strong staining samples, respectively, when CTSB was assessed as a marker. Therefore, CTSB expression might affect metastatic capacity of lung SCC.

In order to further assess their potential prognostic value, IHC and H&E staining was performed to examine CTSB expression in paraffin-embedded tissues (Figure 3A). 99 lung SCC tissue specimens and 29 adjacent normal tissues recruited from the archives of the pathology department were prepared for IHC assay. Among these 99 tumor samples (range 36–72 years), 22 were well differentiated, 35 were moderately differentiated, and 42 were poorly differentiated. As described above, total staining of CTSB was scored as the product of the staining intensity (on a scale of 0–3) × the percentage of cells stained (on a scale of 0–3). As shown in Table 3, in 29 adjacent normal tissues, positive staining of CTSB was rarely detected and total staining score was only 0.59 ± 0.95. However, the other three group including well differentiated, moderately differentiated, poorly differentiated showed a remarkable increasing trend of positive staining of CTSB, with 1.18 ± 1.26, 2.69 ± 1.65, 7.02 ± 1.94 total staining score, respectively. In conclusion, the score of CTSB was paralleled with the increasing severity of epithelial dysplasia. Therefore, over-expression of CTSB was more likely to be present with poor differentiation.

**Table 3:** Cathepsin B immunostaining results of patients with lung SCC

**Tissue type**	**Number**	**Male**	**Female**	**Positive rate **^**b**^	**Intensity **^**b**^	**Staining score**^**a,b**^
Adjacent normal tissues	29	14	15	0.41 ± 0.57	0.52 ± 0.74	0.59 ± 0.95
Well	22	12	10	0.68 ± 0.65	1.00 ± 0.93	1.18 ± 1.26
Moderately	35	16	19	1.49 ± 0.56	1.80 ± 0.47	2.69 ± 1.65
Poorly	42	19	23	2.67 ± 0.48	2.62 ± 0.49	7.02 ± 1.94

^a^ Total staining of CATB was scored as the product of the staining intensity (on a scale of 0–3) × the percentage of cells stained (on a scale of 0–3).

^b^ P value between the four groups: Adjacent normal tissues; Well; Moderately; Poorly.

To assess the correlation between CTSB and the survival rates, 99 patients were retrospectively studied. Total staining of CTSB was still scored as the product of the staining intensity (on a scale of 0–3) × the percentage of cells stained (on a scale of 0–3), resulting in a staining scale of 0–9. The survival data of patients were then classified as weak (0–3), moderate (3.1-6), or strong (6.1-9) staining of CTSB biomarkers. The 5-year survival rates were 41%, 20% and 2% for weak, moderate and strong staining samples, respectively, when CTSB was assessed as a marker (weak VS moderate: P = 0.041; weak VS strong: P = 0.009; moderate VS strong: P = 0.021) (Figure 3B). Multivariate analyses using Cox proportional hazard model also showed that CTSB could also be developed as a prognostic factor for lung SCC.

Analysis of patient data was conducted to study the correlation between CTSB expression and metastatic rates in patients. As showed in Figure 3C, the 2-year metastatic rates were 9%, 21% and 44% for weak, moderate and strong staining samples; the 5-year metastatic rates were 27%, 41% and 71% for weak, moderate and strong staining samples, respectively, when CTSB was assessed as a marker. Therefore, CTSB expression might affect metastatic capacity of lung SCC.

### Down-regulation of CTSB by ShRNA

**Figure 4:**
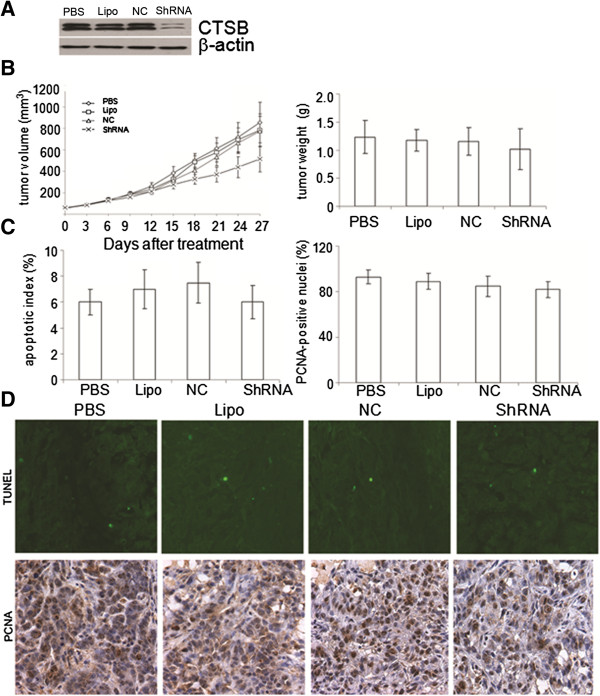
**Effects of suppression of CTSB on tumor xenograft growth in vivo. (A)** The A549 cells were transfected with CTSB-shRNA as described in Materials and Methods. The protein level of CTSB was significantly down-regulated in A549 cells by CTSB-shRNA plasmids. **(B)** Tumor growth curve and tumor weight after treatment with CTSB-shRNA. No statistically significant difference between CTSB-shRNA treated group and the controls was observed (P > 0.05). The animal experiment was repeated three times. **(C & D)** TUNEL assay and PCNA staining. Suppression of CTSB showed no difference in cell proliferation and apoptosis *in vivo*. CTSB-shRNA treated tumor did not show significantly higher percentage of TUNEL-positive nuclei than tumors treated with PBS, Lipo, or NC (6 ± 1.3 versus 6 ± 1, 7 ± 1.5, or 7.5 ± 1.6, 6 ± 1.6) (P > 0.05). The rate of PCNA-positive nuclei in the four groups reached 93.4 ± 6.42, 89.6 ± 7.09, 85.6 ± 9.73, 82.0 ± 7.13 for PBS, Lipo, or NC, CTSB-shRNA, respectively (P > 0.05).

Significant suppression of CTSB expression in CTSB-shRNA treated A549 cells was observed in 48 h. Western blotting showed its expression could be reduced by 89.7% compared with controls (Figure 4A).

### CTSB could not inhibit tumor xenograft growth *in vivo*

Tumor volumes were measured every 3 days during treatment duration until animals were sacrificed. No significant differences in tumor growth were observed during treatment, as shown by the tumor volume and weight (Figure 4B). At the termination of the experiment, tumor volume in the four groups reached 853.401 ± 187.3, 782.39 ± 153.1, 771.904 ± 139.2, 373.078 ± 82.1 mm^3^ for PBS, Lipo, or NC, CTSB-shRNA, respectively (P > 0.05). The weight of tumor treated with CTSB-shRNA also showed no significant differences compared with controls. These data indicated that no statistically significant difference between CTSB-shRNA treated group and the controls was observed in tumor volume, tumor weight, though tumor volume and weight were slightly smaller in the CTSB-shRNA treated group.

### Suppression of CTSB showed no difference in cell proliferation and apoptosis *in vivo*

To address tumor biological processes affected by CTSB-shRNA, we investigated proliferation and apoptosis *in vivo* by PCNA IHC analysis and TUNEL assay (Figure 4C). CTSB-shRNA treated tumor did not show significantly higher percentage of TUNEL-positive nuclei than tumors treated with PBS, Lipo, or NC group (6 ± 1.3 versus 6 ± 1, 7 ± 1.5, or 7.5 ± 1.6, 6 ± 1.6, P > 0.05). The rate of PCNA-positive nuclei in the four groups reached 93.4 ± 6.42, 89.6 ± 7.09, 85.6 ± 9.73, 82.0 ± 7.13 for PBS, Lipo, NC group and CTSB-shRNA, respectively (Figure 4D). Thus, no statistically significant difference between CTSB-shRNA treated group and the controls was observed in PCNA IHC and TUNEL assay.

### Reduced metastatic nodules and prolonged survival in mice bearing experimental lung metastatic tumors by CTSB-shRNA

**Figure 5:**
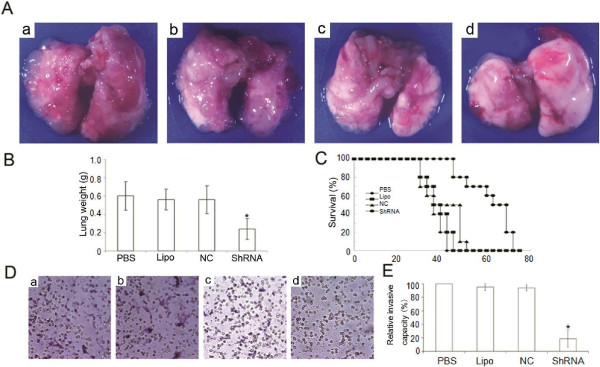
**CTSB inhibited lung metastases *in vitro and in vivo*. (A)** The number of lung metastatic nodules was dramatically reduced in CTSB-shRNA-treated mice compared with controls (a: PBS; b: Lipo; c: NC; d: CTSB-shRNA). **(B)** The lung weight of mice reached 0.6 ± 0.158, 0.56 ± 0.114, 0.56 ± 0.152, 0.24 ± 0.114 for PBS, Lipo, NC, and CTSB-shRNA, respectively (P < 0.05). The animal experiment was repeated three times. **(C)** Kaplan-Meier survival curves of tumor-bearing mice demonstrated the treatment of CTSB-shRNA prolonged the survival of mice with lung metastasis (P < 0.01). **(D** &**E)** CTSB-shRNA was effective in decreasing the invasive capacity of lung cancer cells (a: PBS; b: Lipo; c: NC; d: CTSB-shRNA). The invasive capacity of lung cancer cells decreased nearly 80% after treatment with CTSB-shRNA by quantitative analysis (P < 0.05).

The migratory and invasive properties of cancer cells are crucial to tumor progression. We next investigated whether CTSB-shRNA could inhibit metastatic tumors in the lungs. As shown in Figure 5A, B, metastatic nodules and lung weight were obviously reduced in CTSB-shRNA treated mice. The lung weight reached 0.6 ± 0.158, 0.56 ± 0.114, 0.56 ± 0.152, 0.24 ± 0.114 for PBS, Lipo, NC and CTSB-shRNA, respectively (P < 0.05). Meanwhile, the treatment of CTSB-shRNA prolonged the survival of mice with lung metastasis (P < 0.01) (Figure 5C). The results above demonstrated CTSB influenced the metastatic capacity of lung cancer cells.

### Suppression of CTSB remarkably decreased the invasive capacity of lung cancer cell *in vitro*

After treated with PBS, Lipo, NC and CTSB-shRNA, the invasive capacity of A549 cells was determined by the matrigel invasion assay. The results showed that the invasive capacity of lung cancer cells decreased nearly 80% after treatment with CTSB-shRNA by quantitative analysis (Figure 5D, E).

### Up-regulation of CTSB, Shh and Ptch in metastatic lung SCC

**Figure 6:**
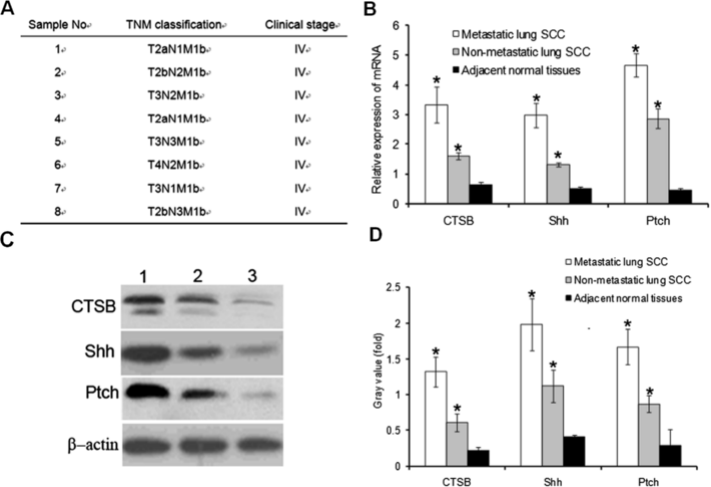
**Up-regulation of CTSB, Shh and Ptch in metastatic lung SCC. (A)** Detailed information of the patients with metastatic lung SCC. **(B)** The mRNA expression level of CTSB, Shh and Ptch in metastatic lung SCC was significantly higher compared with non-metastatic lung SCC and adjacent normal tissues (p < 0.05). **(C** &**D)** The protein expression level of CTSB, Shh and Ptch in metastatic lung SCC was significantly higher compared with non-metastatic lung SCC and adjacent normal tissues. β-actin was used as a loading control (P<0.05).

The metastatic lung SCC specimens were diagnosed histological after staining with H&E, and the clinical stage was determined according to the TNM classification system of the International Union against Cancer. Detailed information of the patients was shown in Figure 6A. Real-time quantitative RT-PCR and western blotting analysis were conducted to examine the expression level of CTSB, Shh and Ptch. As shown in Figure 6B, the mRNA expression level of CTSB, Shh and Ptch in metastatic lung SCC were significantly higher compared with non-metastatic lung SCC and adjacent normal tissues (p < 0.05). Furthermore, the protein expression of CTSB, Shh and Ptch in metastatic lung SCC were significantly higher compared with non-metastatic lung SCC and adjacent normal tissues (p < 0.05) (Figure 6C, D). This data suggested that hedgehog signaling might be activated in metastatic lung SCC, which could affect expression of CTSB that could promote cancer cell invasion.

## Discussion

Lung SCC, one of the most common malignancies worldwide, remains a major health problem with increasing incidence rates even to date [1,2]. Although improvements in surgery, radiotherapy and chemotherapy were made, the survival rate of lung SCC remains low [2]. Thus, there is an urgent to identify novel prognostic and therapeutic biomarkers for lung SCC. In the past, many methods were used to find new tumor biomarkers. Some studies used DNA or mRNA-based technologies, but there are several reasons to conduct a protein-based approach for the identification of potential tumor biomarkers. Proteins are more diverse than DNA or RNA and are responsible for the complexity in a biological system. Alternative splicing and more than 100 post-translational modifications contribute to approximately 100 different proteins derived from a single gene [5,22].

In this study, 2-DE and ESI-Q-TOF MS/MS-based proteomic approach was used to identify differentially expressed proteins between lung SCC and adjacent normal tissue. 31 proteins with significant alteration were identified and these proteins functioned in diverse biological processes. Previous proteomic studies that mainly consisted of Caucasian have identified some key proteins in lung SCC, such as HSP47, cytokeratin 6, cytokeratin 16, and cytokeratin 17 [5]. These proteins are candidate biomarkers for the improvement of lung SCC diagnosis and elucidation of the biology of lung SCC. However, some proteins we found differed from previously identified proteins (Such as ANXA4, CFHR2, CHP, CTSB and so on), which may result from ethnic differences or tumor heterogeneity.

Among these identified proteins, CTSB was up-regulated 5.0-fold in tumor compared with pair adjacent normal tissue (P < 0.05). Furthermore, western blotting also showed similar results. In addition, IHC showed over-expression of CTSB was more likely to be present with poor differentiation of lung SCC. Analysis of clinical data displayed over-expression of CTSB was correlated with poor prognosis and increased incidence of distant metastases.

Increased levels of CTSB have been reported in several cancers, but relatively little is known about CTSB’s involvement in lung cancer proliferation and apoptosis. In this study, data of animal experiment indicated that no statistically significant difference between CTSB-shRNA treated group and the controls was observed in tumor volume, tumor weight, TUNEL assay and PCNA IHC. Thus, CTSB cannot directly affect proliferation and apoptosis of lung cancer cells.

The migratory and invasive properties of cancer cells are crucial to tumor progression. We next investigated whether CTSB-shRNA could inhibit lung metastatic tumors. CTSB-shRNA reduced lung metastatic nodules and prolonged survival in mice bearing experimental lung metastatic tumors. Matrigel invasion assay showed the invasive capacity of lung cancer cells decreased nearly 80% after treatment with CTSB-shRNA.

The hedgehog pathway regulates fundamental biological processes such as stem cell maintenance, cell differentiation, tissue polarity, and cell proliferation [23]. Inappropriate activation of hedgehog signaling pathway has been implicated in the development of a variety of cancers, such as breast cancer, prostate cancer, lung cancer and so on [24-26]. It has been reported that CTSB was a downstream target of hedgehog signaling in breast cancer and hedgehog signaling activated CTSB was associated with tumor invasion [27]. In this paper, we attempted to investigate the expression level of Shh and Ptch, two key proteins of hedgehog pathway, in metastatic lung SCC tissues. mRNA or protein expression level of Shh, Ptch and Ptch CTSB in metastatic lung SCC were significantly higher compared with non-metastatic lung SCC and adjacent normal tissues. Taken together, it suggested that hedgehog signaling might be activated in metastatic lung SCC, which could affect expression of CTSB that could promote cancer cell invasion.

## Conclusions

In this study, comparative proteomic analysis of lung SCC and the adjacent normal tissues revealed 31 differentially expressed proteins. Dysregulation of CTSB was associated with aberrant metastatic capacity. Molecular mechanism analysis suggested hedgehog signaling might be activated in metastatic lung SCC which could affect the expression of CTSB that could influence the invasive activity of lung SCC cell. As a result, CTSB might be a promising prognostic and therapy marker for human lung SCC.

## Competing interests

The authors declared that they have no competing interests.

## Authors’ contributions

FMG, XCP, CL, GBS, CJZ, ZLQ, LHL and YXS performed all of the experiments. YWZ collected patient tissues. FMG, XCP, CL, GBS, XZ participated in its design and wrote the manuscript. All authors read and approved the final manuscript.
